# Immune metabolic restoration in systemic lupus erythematosus: the impact of gut microbiota, probiotics, and nutritional synergy

**DOI:** 10.3389/fimmu.2025.1602235

**Published:** 2025-06-04

**Authors:** Douae Nihed Habiballah, Fanzhu Li, Lai Jiang

**Affiliations:** School of Pharmaceutical Sciences, Zhejiang Chinese Medical University, Hangzhou, China

**Keywords:** systemic lupus erythematosus, probiotics, gut microbiota, vitamin D, 6-gingerol, immune modulation

## Abstract

Gut microbiota alterations have been considered one of the attributes of Systemic Lupus Erythematosus (SLE), and may offer an immunological disorder and even cause the disease. The probiotic administration, especially the *Lactobacillus* and *Bifidobacterium* probiotics, is becoming highly utilized for the maintenance of the intestinal barrier’s integrity and immune function, minimizing further the risks of developing some complications such as lupus nephritis, mechanisms that have remained so incompletely defined. This review focuses on the analysis of literature data about the influence of certain probiotic strains on the pathogenesis and course of SLE as immunomodulators and a new therapy strategy that seeks to achieve a synergistic effect with the help of an additional probiotic in combination with dietary supplements gingerols or vitamin D. The current review, therefore, provided the limitations in current trials addressing SLE and therapy optimization. The work is done with the intention of addressing the existing gaps in knowledge, thereby creating more space for new approaches to emerge toward the SLE management and improvement of patients’ outcomes.

## 1 Introduction

Systemic lupus erythematosus (SLE) is a multifaceted autoimmune disorder that includes loss of tolerance to self-antigen and subsequent activation of autoreactive T and B cells, production of dysregulated cytokines (1), and the production of autoantibodies such as anti-phospholipid antibodies (anti-aPL) (2), anti-ribonucleoprotein antibodies (anti-RNP) (3), anti-double-stranded DNA (anti-dsDNA) (4), and anti-IgE (5), and circulating immune complexes which when deposited in organs activate complement cascades leading to tissue injury (6). The general etiological factors playing a role in the causation of the disease are hormonal imbalance, alcohol consumption, exposure to UV radiation, genetic susceptibility, and smoking the latter is dose-response risk factor (7). Other risks include silica, diets containing canavanine, viral infections, vitamin D deficiency, and alfalfa sprouts (8). Drug-induced lupus has been associated with over 100 medications, especially procainamide and hydralazine, which alter self-antigens and lead to DNA demethylation, mimicking lupus symptoms (9). SLE may thus affect several systems, with notable complications including those of lupus nephritis and gastrointestinal vasculitis originating from the actions of the deposition and its resultant inflammation produced (10, 11). Globally, SLE prevalence is about 43.7/100,000 patients with the disease spread at a higher rate among women (78.73/100,000) compared to men (9.26/100,000). The prevalence is 50.37/100,000 in Chinese population estimated to affect 622,526 women and 76,677 men (12).

The available treatment methods in SLE are chiefly immunosuppressants. Certainly, these therapies are effective, but they most often lead to severe long-term complications such as infections, Secondary malignancy, ovarian failure, disturbances in gastrointestinal tract functionality, miscellaneous hematologic conditions that include leucopenia or anemia, organ damage, and long-term use of corticosteroids may lead to weight gain, delivery of osteoporosis, and diabetes mellitus (13–15). The recently developed insights also appear to indicate that dietary interventions, more particularly those that modify gut flora, may yield novel therapeutic avenues (16). Probiotics are living microbes that offer the host a beneficial effect on health (17), boosting immunity and aiding in the healing of autoimmune diseases like SLE (18). Besides, some nutritional constituents, such as ginger and vitamin D supplements, have been observed to offer anticatabolic and immunomodulatory effects (19, 20).

Literature reviews regarding the association of probiotics with lupus indicate that they may have beneficial contributions to immune regulation and inflammation, two pivotal components in lupus pathogenesis. Few of them are identified with showing that probiotics regulate gut microbiota to an extent that may help improve symptoms of lupus and overall health. However, most of the current evidence is limited and inconsistent. Further research is thus necessary to establish the exact mechanisms and to provide clear treatment guidelines for lupus patients. This literature review aims to discuss the interrelationships among gut flora, probiotics, vitamin D, and 6-gingerol in relation to SLE. We will review the use of probiotics in mitigating SLE symptoms, the potential additional benefits of co-supplementation with either ginger or vitamin D, and the observed gender disparities in disease prevalence. This review aims to consolidate existing research findings to determine prevailing gaps in knowledge and propose future research directions that might be done to have a deeper comprehension of SLE management.

## 2 Gut flora and systemic lupus erythematosus

Human intestinal microbiota composed of trillions of microbials is a key player in maintaining numerous physiological processes, including immune status, metabolism, and health. There has been focus on recent investigations identifying the participation of gut flora in autoimmune illness, most importantly SLE, while alteration of the gut microbiome or “dysbiosis,” has been suspected of disease causation. This section addresses altered gut microbiota in SLE patients, its contribution to disease progression, and gender-specific differences in microbial populations that may be responsible for the higher prevalence of lupus in females. Clarification of these associations may yield novel therapeutic and preventive measures.

### 2.1 Gut flora

Bacteria, yeast, and fungi are microorganisms that together are referred to as gut flora, gut microbiota, or gut microbiome. Gut microbiota is responsible for the basic health in a human being. The gut is inhabited by approximately 100 trillion of these microorganisms, primarily found in the large intestine with a smaller amount in the final part of the small intestine. These gut bacteria thrive on the byproducts of digestion (21). The human body is estimated to have over a thousand different microbial species, which can account for 54.7% to 55% of fecal weight (22).

Gut microbiota contributes significantly to multiple functions (**Table 1**). It is first an immune system supporter; around 80% of the body’s immune cells live in the gut, where the microbiota helps trigger immune reactions and protect the body from pathogens (23, 24). Second, gut microbiota keeps metabolic health by creating essential vitamins and nutrients. They are involved in the functions of the liver, insulin sensitivity, control of appetite, and weight regulation, and also aid in the bile metabolism beneficial in the digestion of fat (25, 26). Furthermore, a balanced gut microbiota may lower the likelihood of illnesses like type 2 diabetes and other metabolic disorders (27, 28). More and more, the connection between brain and gut health is also being realized. Gut microbiota is responsible for central nervous system function and, perhaps, mood and cognitive status (29–31).

**Table 1:** Studies on Gut Microbiota in SLE.

Strains	Population	Key Findings
Gut microbiota	*MRL/lpr* mice	The study found that gut microbiota composition changes with the severity of lupus, potentially influencing disease progression (34).
Gut microbiota	SLE patients	Hevia et al. identified gut microbiota dysbiosis in SLE patients, characterized by reduced Firmicutes and increased Proteobacteria, which may disrupt SCFA production and correlate with disease activity, suggesting gut microbiota as a therapeutic target (35).
Gut microbiota	SLE patients *NZB/W F1* mice	According to the study, SLE patients with active illness and lupus-prone mice exhibited an altered gut microbiota, characterized by less diversity and more abundance of Gram-negative bacteria. Specific genera such as *Odoribacter* and *Blautia* were identified as differing between SLE patients and healthy controls (46).
Gut microbiota	SLE patients	The study found associations between gut flora and the disease onset, course, activity, and therapeutic effects in SLE patients (47).
Gut microbiota	SLE patients	SLE patients exhibit gut microbiota dysbiosis, marked by a reduced presence of Firmicutes and an elevated presence of Proteobacteria. This imbalance may impact SCFA production, which is crucial for gut health and immunological control. Specific microbial shifts correlate with disease activity, suggesting that gut microbiota may represent a viable treatment target for SLE (48).
*B. fragilis*	*MRL/lpr* mice	*B. fragilis* mitigated lupus nephritis symptoms by modulating CD1d and CD86 expression in B cells (49).
Gut microbiota	*MRL/lpr* mice	Alterations in gut flora contribute to the control of lupus nephritis (50).

Dysbiosis, however, can lead to pathological states like inflammation and autoimmunity (24, 32). Several studies have presented evidence of deranged gut microbiota and the associated disorder like lupus (33, 34). Therefore, it is well to have sufficient diversity in the gut microbiota for overall health (25).

### 2.2 Characteristics of the microenvironment of the gut flora in SLE patients

Research has revealed significant differences in gut flora among healthy and SLE patients. Gut microbiota in healthy individuals is generally rich and diverse, undertaking a range of body functions, including immune regulation and metabolic balance. In contrast, individuals with SLE have reduced diversity of gut microbiota, with a structural and functional disorder known as dysbiosis (35). Studies indicated that SLE patients have some of the populations of bacteria depleted or altered, which initiate inflammatory process development and immune dysregulation. The imbalance is generally the depletion of beneficial bacteria. In a healthy gut, *Firmicutes* and *Bacteroidetes* together account for approximately 98% of the bacterial community, low F/B ratio is linked to gut microbiota dysbiosis, once more pointing to microbial imbalance as being key to SLE pathogenesis (33, 36). In Kim et al.’s model, microbial populations were distinct from pre-disease, and lupus-like phenotype development was linked to extreme decreases in the diversity of the gut bacteria (37).

SLE patients are usually characterized by a condition called leaky gut, which is compromised integrity of the intestinal barrier. The compromised integrity permits numerous pathogens, such as microbes, to cross the mucosal layer of the intestine. This invasion is responsible for killing and injuring the intestinal cells, particularly goblet cells and Paneth cells. Both are extremely important to maintain gut integrity: Paneth cells induce defense and control microbes, while goblet cells produce the protective layer of intestinal mucus. The loss of these cells can further exacerbate the intestinal barrier dysfunction, creating a vicious cycle that may contribute to the overall pathology observed in SLE patients (35, 38).

In SLE patients, the gut microenvironment can experience a heightened level of inflammation brought about by the autoimmune nature of the disease. Such an inflammation has the ability to significantly alter the gut bacteria composition and also their function. The changes in the gut microbiota have the potential to influence immune system pathways and become key contributors to SLE pathology. For instance, dysbiosis has the potential to lead to T cell population alterations, which in turn may produce a tipping in the balance towards systemic autoimmunity. Dysregulation will persist to enhance autoreactive T cell activation and lead to disease progression and exacerbation (39).

Changes in the gut microbiota can significantly impact the production of short-chain fatty acids (SCFAs) and other metabolites which in turn modulate systemic inflammation and immunity. SCFAs, including propionate, acetate, and butyrate, strongly modulate inflammation, supporting gut health and immune function. With dysbiosis of the gut microbiota, synthesis of such beneficial metabolites could be reduced, which may promote increased inflammation and compromised immune function. Such disturbance may exacerbate autoimmune conditions like SLE, as defective SCFA generation might be responsible for intestinal wall disruption and modulation of immune signaling pathways, thereby leading to disease pathology (39).

### 2.3 Sex-based differences in gut microbiota and their implications for SLE

Mounting evidence indicates that the structure of gut microbiota differs drastically in women and men as a result of hormones, lifestyle, and diet (40, 41). Women possess higher levels of some beneficial bacterial species, whereas microbial diversity is higher in men (42, 43). These variations affect sex based immune modulation and disease susceptibility (44).

SLE is a disease that primarily targets women, making it one of the known sex-specific autoimmune diseases. The study of gut microbiota in *Murphy Roths Large/Lymphoproliferative (MRL/lpr)* lupus-prone mice discovered striking sex-dependent differences. The female MRL/lpr mice have lower Lactobacillaceae and higher Lachnospiraceae than the control group. Male MRL/lpr mice do not show any significant differences in gut microbial composition from controls. Likewise, in control groups, female mice have higher abundance of Lactobacillaceae and Streptococcaceae, with lower abundance of Lachnospiraceae and Clostridiaceae than males. In lupus-prone females, reduced abundance of Erysipelotrichaceae and Bifidobacterium, with increased Lachnospiraceae and Bacteroidetes S24–7 abundance, has also been reported (44).

These findings suggest that microbiological dysbioses specific to sex, more precisely the increased prevalence of Lachnospiraceae in females, may account for the earlier onset and greater severity of lupus symptoms. The structure of gut microbiota in female lupus-susceptible mice appears to favor inflammatory processes, which can promote disease onset (44).

Sex hormones, particularly estrogen and testosterone also play a central role in the determination of gut microbiota and SLE susceptibility. Estrogen enhances B cell activation and antibody secretion, leading to increased immune responses, while testosterone exert immunosuppressive effects through inhibition of antibody production. This hormonal effect accounts for sexual characteristics changes in immune function, and the higher susceptibility of women to autoimmune diseases like SLE (45).

It will be crucial to distinguish the interplay of gut microbiota and the sex hormones as it can potentially unlock the mechanisms of higher susceptibility of females to SLE and also unveil novel avenues of therapeutic intervention by manipulation of the gut microbiome for controlling disease course (45).

## 3 Probiotics and systemic lupus erythematosus

Probiotics are live microorganisms significant for their role in immunomodulation in SLE patients, as they restore gut microbiota balance and enhance immune function. Studies have shown that probiotics support gut barrier function, modulate immune response and reduce inflammation (51). Strains such as Lactobacillus and Bifidobacterium affect immune pathways, and probiotic metabolites, including SCFAs, further contribute to immune regulation (52). This section will explore how probiotics affect immune cells and inflammatory mediators and their potential synergistic effects with nutritional treatments for the management of SLE.

### 3.1 Probiotics’ mechanism of action

**Figure 1:**
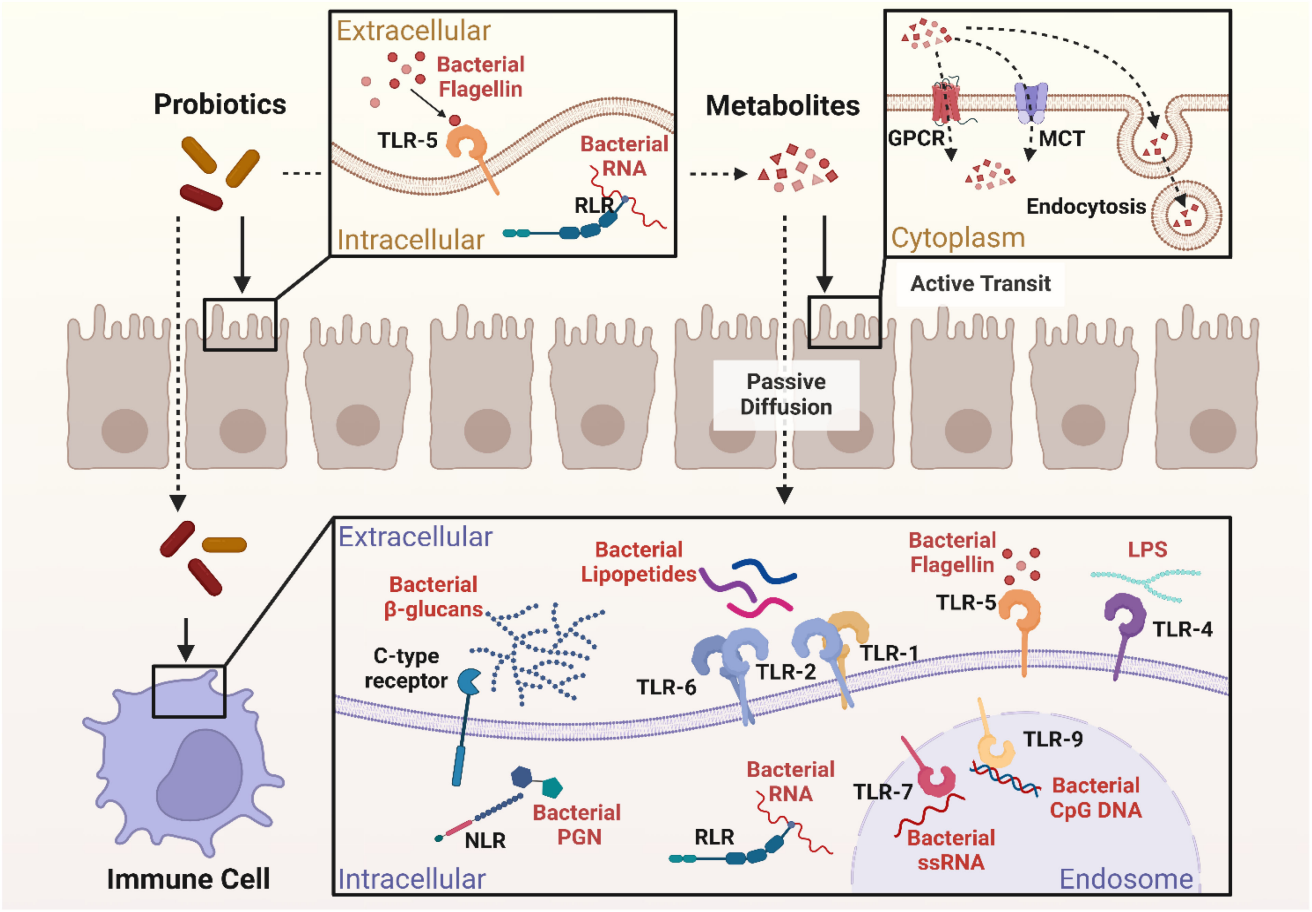
Interactions between probiotics, epithelial cells, and immune cells. Created in BioRender. Jiang, L. (2025) https://BioRender.com/l8zjnxu.

Through interaction with immune cells and epithelial cells, probiotics participate in the control of immunological response (**Figure 1**). Probiotics act on epithelial and immune cells in two manners: one is direct probiotic contact with epithelial and immune cells via microbe-associated molecular patterns (MAMPs) such as microbial conserved molecules like surface proteins (SLPs), Lipoteichoic acid (LTA), flagellins, pilins, capsular polysaccharides (CPSs), peptidoglycan (PGN) and lipopolysaccharides (LPS), which may be sensed by host immune receptors to mediate such interactions (53–57). The other is an indirect interaction through the release of metabolites, including SCFAs, which can influence physiological responses through different signaling pathways (58).

#### 3.1.1 Direct contact

The close interaction between host cells and probiotics is through the recognition of MAMPs by pattern recognition receptors (PRRs) on the host cell surface. MAMPs are identified by various types of PRRs, which play a crucial role in stimulating immune responses (53, 54). A few of the significant PRRs are NOD-like receptors (NLRs), which are intracellular receptors present both in immune cells and epithelial cells. They identify microbiological components and stimulate immune responses (59).

Toll-like receptors (TLRs) are another important group of PRRs found on the cell surface or in endosomal compartments of many immune and epithelial cells. TLRs allow the immune system to detect pathogens under various conditions. They are capable of sensing various microbiological components such as PGN in the cell walls of Gram-positive bacteria (57), LTA found in Gram-positive bacteria like *lactobacillus rhamnosus (L. rhamnosus)* (54, 57), and LPS in Gram-negative probiotics like *Escherichia coli (E.coli)* Nissle 1917 (57). Other components, such as flagellin and Unmethylated cytosine-phosphate-guanine DNA (CpG DNA), are also present in certain probiotics and recognized by TLRs (57, 60).

C-type lectin receptors on immune cells, including dendritic cells, neutrophils, monocytes and macrophages, have the ability to recognize carbohydrates (e.g., Propionibacteria) derived from probiotics and fungi (53, 61, 62). Additionally, RIG-I-like receptors (RLRs) within the immune and epithelial cell cytoplasm also possess the ability to recognize Ribonucleic Acid (RNA), which is normally viral but may also be able to detect probiotic RNA in specific situations (59, 63).

These PRRs are expressed on the surface of dendritic cells, macrophages, and many other immune cells, and their activation could be done through the help of some strains of probiotics which enables different signaling pathways through the production of cytokines and chemokines that, together, regulate inflammation, enhance barrier integrity of the intestine, and maintain overall gut health in a homeostatic state (59).

#### 3.1.2 Indirect effects

Indirect probiotic interaction with epithelial cells involves a variety of metabolites such as exopolysaccharides, bile acid derivatives, and SCFAs, which play significant roles in the preservation of intestinal homeostasis, immune system regulation, and strengthening of the epithelial barrier. These metabolites are capable of exerting passive and active effects on epithelial cells and therefore to overall gut health (64).

Small molecule hydrophobic metabolites such as propionate, acetate, and butyrate can cross the epithelial barrier by simple diffusion along a concentration gradient from zones of elevated concentration in the intestinal lumen to places of diminished concentration in the epithelial cells (65). On the other hand, SCFAs such as acetate, butyrate and propionate can also rely on monocarboxylate transporters for active transport, which ensures targeted and controlled uptake of metabolites (66). Nevertheless, for large probiotic-derived molecules, such as extracellular polysaccharides (ECPS) or extracellular vesicles (EVs), endocytosis is used as a receptor-mediated mechanism of active transport (67, 68).

SCFAs also signal through G protein-coupled receptors (GPCRs) on epithelial cells, such as GPR41 and GPR43, by which they activate intracellular signaling pathways to take up (69). These interactions indirectly modulate various immune signaling pathways, especially RLRs- and TLRs-mediated signaling pathways. SCFAs are able to regulate the expression of RLRs or downstream signaling and modulate host antiviral and anti-inflammatory response, thus being involved in intestinal inflammation and regulation of immunity (64).

Probiotics interact with epithelial and immune cells through microbial patterns and metabolites. These interactions enhance gut barrier integrity, modulate inflammation, and support immune homeostasis, highlighting the therapeutic significance of probiotics in autoimmune diseases such as SLE.

### 3.2 Regulation of intestinal structure and function by probiotics

Dysbiosis, or gut microbiota disturbance, is a compositional and functional change contributing greatly to the basic pathogenesis of SLE. Dysbiosis destroys the integrity of intestinal barriers, thus making the mucosa permeable to pathological organisms and leading to cell death among small intestinal cells such as Paneth and goblet cells (33, 70, 71). There is evidence in MRL/lpr mice that higher levels of LPS are associated with impaired intestinal barrier function (50). Zonula Occludens-1 (ZO-1) and occludin are two tight junction proteins in the intestinal barrier that are disrupted by LPS. For instance, Guo et al. (72) showed that LPS increases intestinal permeability via induction of expression of TLR4 and CD14 in enterocytes, an event that is mechanistically related to destabilization of tight junctions. These findings demonstrate one way in which LPS could be implicated in gut barrier breakdown in autoimmune conditions, such as lupus (72).

Probiotic-derived metabolites have a protective role in ensuring the intestinal epithelial barrier. They exert their effects either directly by stimulating goblet cell-stimulated mucus secretion such as mucin 2 (MUC2) secretion in colonic epithelial cells that enhances intestinal barrier function and immune response. Probiotic induction of mucin secretion offers not only a shield over the gut lining itself but also immune-modulating effects, therefore overall enhancement of gut health (67, 73, 74), or by interacting with specific receptors, which further stimulate the release of antimicrobial peptides or increase tight junction protein expression (53, 75, 76). For instance, an experiment on the effect of probiotics on the structure of tight junction utilized the marker claudin-7 and observed that *Lactobacillus plantarum (L. plantarum)* and *Akkermansia muciniphila (A. muciniphila)* maintain gut barrier integrity and function in a model of SLE (77).

Another study revealed that Probiotics such as *L. rhamnosus* NK210, *Lactococcus lactis* NK209 *(Lc. lactis)* and *Bifidobacterium longum* NK219 *(B. longum)* has improved tight junction protein levels and inhibited LPS synthesis by gut bacteria. Thereby ameliorating the condition of leaky gut in mice with inflamed intestines (78). Furthermore, Mengchen et al.’s investigation, which examined fecal DNA from experimental groups both before and after probiotic treatment, found that *A. muciniphila* and *L. plantarum* considerably changed the gut microbiota’s diversity and structure (77).

Such manipulation of gut microbiota by various probiotic supplements could restore the dysbiotic F/B ratio, which is important both in the prevention and treatment of SLE. It’s highly important to note that balanced gut microbiota is achieved when the F/B ratio is equated, and imbalances occur when this ratio becomes either elevated or diminished. Moreover, various probiotics have different effects on the F/B ratio; therefore, the choice and dosage of probiotics should be proportional to induce balance and eubiosis of gut microbiota (79).

Probiotics reinforce the intestinal barrier by stimulating mucus secretion, maintaining tight junction integrity, and inhibiting lipopolysaccharide translocation. This regulation mitigates leaky gut phenomena, which are key contributors to SLE progression, emphasizing their role in gut-centric therapies.

### 3.3 Probiotics promote renal structure and function to prevent lupus nephritis

Inflammation of the gut permits harmful germs to enter the bloodstream and secrete LPS, which stimulates the Nuclear factor kappa-light-chain-enhancer of activated B cells (NF-κB) pathway and enhances pro-inflammatory cytokines and autoantibody production, leading to immune complex generation and deposition in the kidney, which causes organ damage. LPS also activates albumin excretion, which is associated with the aggravation of lupus nephritis (80).

A study of *Lactobacillus* supplementation in *MRL/lpr* mice confirmed that it improves the elimination of LPS by inducing intestinal alkaline phosphatase (IAP) activity, which maintains tight junction integrity and barrier function. IAP dephosphorylates LPS and prevents it from binding TLR4, thus inhibiting NF-κB pathway activation, ultimately reducing proinflammatory cytokine release (50). Moreover, SCFA has proven to inhibit the activation of the NF-κB pathway (81). Weakened tight junction increases the intestinal barrier permeability, which enables leaky gut effect, leading to increased levels of anti-*Ruminococcus gnavus* antibodies (anti-RG) that act on *Ruminococcus gnavus* (RG) antigens and worsen kidney damage in lupus patients (82).

Kidney disease in *New Zealand Black/White* F1 hybrid mice (*NZBWF1* mice) is characterized by hyperblood pressure, albuminuria, and altered urine creatinine levels, in correlation with anti-dsDNA antibody titers. Probiotic *Lactobacillus fermentum* CECT5716 (LC40) prevents lupus nephritis caused kidney damage via lowering systemic inflammation and preserving the balance of the gut microbiota and thereby preventing immune complex deposition within the kidneys. LC40 supplementation reduces autoantibody levels and pro-inflammatory cytokines, indicating broader immunomodulatory effects. These results demonstrate its potential as a lupus nephritis adjuvant treatment (83). Furthermore, butyrate has been shown to improve renal disease (**Table 2**). Through the G-protein-coupled receptor. Butyrate therapy demonstrated effectiveness in reducing kidney damage in lupus-prone mice by boosting the F/B ratio and microbial diversity (84, 85).

**Table 2:** Studies on Probiotics in SLE.

Probiotic Strains	Participants	Dosage	Delivery system	Key Findings
*L. rhamnosus* LC-STH-13	Female *MRL/lpr* mice	Not specified	Oral administration	Ameliorated SLE progression by inhibiting the TLR9/NF-κB pathway, reduced anti-autoantibody levels, and improved kidney inflammation (147).
*L. delbrueckii;* *L. Rhamnosus;* *L. Casei; B. bifidum*	20 patients	Not specified	Oral administration	This study found that the use of probiotics reduced clinical disease activity significantly, as measured by the SLE Disease Activity Index 2000 (SLEDAI-2K). No adverse effects were noted (158).
*L. helveticus* R0052; *B. infantis* R0033; *B. bifidum* R0071; fructo-oligosaccharides.	23 in the synbiotic group; 23 in the placebo group	One capsule/day containing 3 x 109 CFU probiotics + 80 mg FOS, for 60 days	Oral capsule (synbiotic formulation)	The study found significant changes in gut microbiota composition and decreases in inflammatory indicators such as serum Hs-CRP, IL-6, and IL-17, suggesting potential benefits of synbiotics in managing SLE (159).
*L. fermentum* CECT5716 (LC40)	*NZB/W* F1 SLE; *NZW/LacJ* mice	5 × 10^8^ CFU/day for 13 weeks	Oral administration	The study demonstrated an improved renal outcome by restoring immune balance through regulation of the Th/Tregs cell ratio, alongside reducing plasma pro-inflammatory cytokine levels, suggesting its potential in mitigating lupus-associated kidney damage (83).
*A. muciniphila;* *L. plantarum*	*MRL/lpr* mice	Administered every 2 days from 8 to 15 weeks of age (CFU not specified)	Oral administration	*A. muciniphila* and *L. plantarum* alleviated SLE in mice by reducing systemic inflammation, lowering IL-6 and IL-17 levels, elevating IL-10, restoring gut barrier integrity, diminishing renal IgG deposition, and enhancing kidney function, likely through immune modulation and gut microbiota regulation (77).
*L. casei* B255; *L. reuteri* DSM 17509; *L. plantarum* LP299v	*NZB/W* F1 mice	Daily administration of L. casei B255, L. reuteri DSM 17509, or L. plantarum LP299v (CFU not specified)	Oral administration	*L. casei* B255 boosted CD4+ Foxp3+ Tregs, IL-10 production, and B7-1/B7–2 expression on DCs, delaying autoantibody (ANA) emergence; *L. reuteri* DSM 17509 induced milder IL-10 elevation and delayed proteinuria onset, while *L. plantarum* LP299v showed minimal impact on disease progression, survival, and IL-10 levels, highlighting *L. casei* as the most potent immunoregulator (125).
*L. paracasei* GMNL-32; *L. reuteri* GMNL-89; *L. reuteri* GMNL-263	*NZB/W* F1 mice	Dietary supplementation with GMNL-32, GMNL-89, or GMNL-263 (CFU and duration not specified)	Oral administration via diet	The three Lactobacillus strains improved hepatic injuries and reduced inflammation in lupus-prone mice by suppressing MAPK/NF-κB signaling pathways and lowering IL-1β, IL-6, and TNF-α expression (160).
*L. paracasei* GMNL-32; *L. reuteri* GMNL-89; *L. reuteri* GMNL-263	*NZB/W F1* mice	Oral gavage with GMNL-32, GMNL-89, or GMNL-263 (CFU and duration not specified)	Oral gavage	The oral Lactobacillus administration in NZB/W F1 mice enhanced antioxidant levels in serum/liver, suppressed hepatic TLR-4, -5, -7, and -9 expression, reduced pro-inflammatory cytokines (IL-1β, IL-6, TNF-α), and increased splenic CD4+ CD25+ Foxp3+ Treg, alongside elevated Foxp3 mRNA in Tregs (124).
*L. delbrueckii;* *L. rhamnosus*	20 SLE patients	10^7^ CFU/mL L. rhamnosus, 10^5^ CFU/mL L. delbrueckii, or combination; 48-hour incubation	Ex vivo PBMC culture treatment	*L. delbrueckii* and *L. rhamnosus* elevated anti-inflammatory cytokines (IL-10, TGF-β) and reduced pro-inflammatory IL-6 levels in SLE patients (146).
*L. delbrueckii;* *L. rhamnosus*	Sample includes SLE patients and healthy controls; exact number not specified	Co-culture with L. delbrueckii, L. rhamnosus, or combination for 48 hours (CFU not specified)	Ex vivo macrophage culture treatment	*L. delbrueckii* and *L. rhamnosus* reduced pro-inflammatory markers (CD14, CD80, HLA-DR) on macrophages, elevated IL-10 and TGF-β, suppressed IL-12, IL-1β, and TNF-α, and induced an anti-inflammatory macrophage phenotype in SLE patients (101).
*L. delbrueckii;* *L. rhamnosus*	Lupus patients and healthy controls	Treatment with L. delbrueckii, L. rhamnosus, or combination (CFU and duration not specified)	Ex vivo treatment of monocyte-derived dendritic cells	Tolerogenic probiotics inhibited migratory potential of lupus patient-derived DCs by downregulating inflammatory chemokine receptors (CXCR3, CCR5, CCR4, CCR3), while enhancing regulatory DC traits through elevated indoleamine 2,3-dioxygenase (IDO) and IL-10 alongside reduced IL-12 (108).
*L.rhamnosus* GG ATCC9595; *L.delbrueckii* PTSS 1743 DSM 20072; prednisolone	*BALB/c* mice	Oral administration of L. delbrueckii, L. rhamnosus, or combination (CFU and duration not specified)	Oral administration	The study demonstrates their impact to reduced anti-dsDNA, ANA, and anti-RNP antibodies, alleviated proteinuria and disease severity, boosted CD4+ CD25+ FoxP3+ Tregs, lowered IL-6, and elevated IL-10 and TGF-β, collectively ameliorating SLE symptoms in pristane-induced lupus mice (134).
Tolerogenic *Lactobacillus* probiotics, specifically *L. rhamnosus* and *L. delbrueckii*.	*BALB/c* mice	Oral administration of L. delbrueckii, L. rhamnosus, or combination (CFU and duration not specified)	Oral administration	Tolerogenic *Lactobacillus* probiotics reduced Th1/Th17 cell populations, elevated Tregs and IL-10, suppressed pro-inflammatory cytokines (IL-17, IFN-γ) and autoantibodies (ANA, anti-dsDNA, anti-RNP), diminished lipogranuloma mass, and delayed SLE progression in pristane-induced lupus mice (120).
Association of Tacrolimus and *L. acidophilus*	*MRL/lpr* mice	5 mg/kg of Tac and/or 50 mg/kg of LA daily for 8 weeks.	Oral administration	*L. acidophilus* supplementation increased Tregs, reduced Th17 cells, and lowered serum anti-dsDNA levels, enhancing tacrolimus efficacy by restoring Th17/Treg balance via the SIGNR3 pathway, thereby improving immune regulation (37).

An investigation by Kim et al. indicated that the combination treatment between Tacrolimus (Tac) and *Lactobacillus acidophilus* (*L. acidophilus*) reduces kidney pathology scores and serum levels of immunoglobulin G2a and anti-dsDNA antibodies (37). Furthermore, it has been shown that *A. muciniphila* and *L. plantarum* both lessen kidney damage by lowering proteinuria, creatinine, and urea nitrogen levels (77).

### 3.4 Immune modulatory mechanisms of probiotic in SLE

Early on in the inflammatory process, changes in innate immune cells within the intestines have been observed, including mast cells, dendritic cells, neutrophils, and macrophages. These immune cells are recruited due to self-antigens and changes in the composition of commensal bacteria, a process termed dysbiosis. This dysbiosis often leads in turn to colonic barrier dysfunctionalities, a condition notoriously known as “leaky gut” (86, 87). The colonic barrier breakdown thus allows microbial derivation products, whose most common representatives should be considered LPS and Teichoic acid (TA), to begin to translocate into the layer underneath the epithelium, the lamina propria, launching an immune challenge that will express itself through numerous pro-inflammatory cytokines and complex-associated cascades (82, 88).

Chronic inflammation seen in SLE is a result of pro-inflammatory innate immune cells being recruited and activated, leading to the activation of the adaptive immune response, which culminates in autoantibody production. Both the innate and adaptive responses have a central role in the chronic tissue injury and epithelial damage that represent SLE (89, 90).

**Figure 2:**
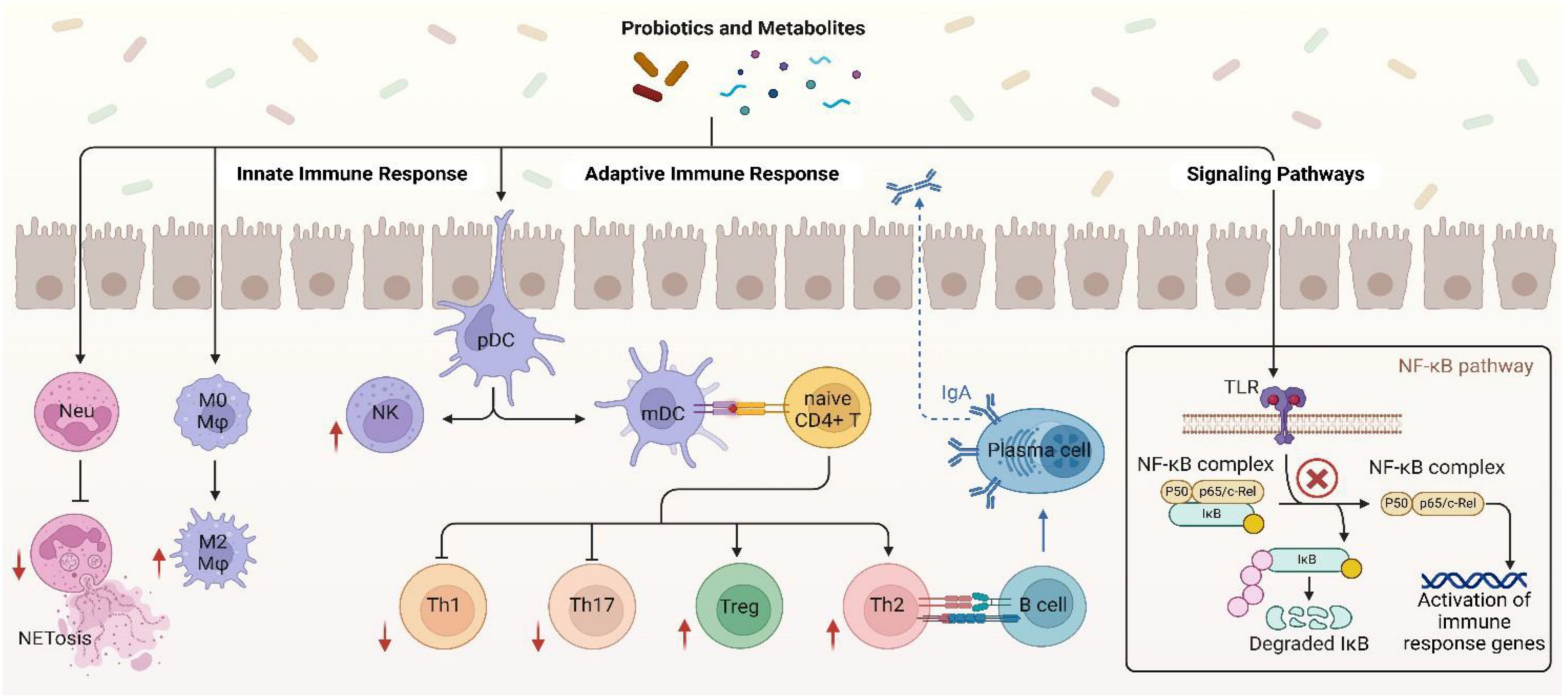
Immune modulatory mechanisms of probiotic in SLE. Created in BioRender. Jiang, L. (2025) https://BioRender.com/smgamxw.

Probiotics have been shown to have immunomodulatory effects that are particularly intriguing in relation to controlling the immunological responses in individuals with SLE (88). Numerous metabolic products produced by probiotics may have an impact on immunocompetent cells and the signal pathways that are connected to them in the pathophysiology of SLE (**Figure 2**) (87). The manner in which probiotics affect both innate and adaptive immune cells, including cytokine production and the modulation of some crucial signal pathways, has been the subject of much research up to this point (82, 87).

#### 3.4.1 Probiotics impact on innate immune response

Probiotics, especially Lactobacillus, have been shown to be able to attain the remarkable capability of enhancing neutrophil functionality by secreting exopolysaccharides (EPS) that activate the TLR2/MyD88 signaling pathway, thereby facilitating chemotaxis and phagocytosis. Such cells will be in a better position to engulf and digest pathogens effectively (91). Other studies also identified that some probiotics are able to modulate Neutrophil Extracellular Trap formation (NETosis) (92), a mechanism by which neutrophils capture and kill pathogens by the release of Neutrophil Extracellular Traps (NETs). This mechanism is relevant in SLE, where there is excessive NETosis that acts to promote injury and disease development (93). Probiotic strains such as *L. rhamnosus* GG have shown the ability to inhibit NET production stimulated by phorbol 12-myristate 13-acetate (PMA) and *Staphylococcus aureus* (*S. aureus*), along with antioxidative activity that inhibits generation of reactive oxygen species (ROS) via TLR2-dependent mechanisms and consequently decreases cytotoxicity of the host cells (**Figure 2**) (92).

According to reports, some probiotic strains have the ability to eliminate intracellular infections by triggering macrophage activation to the M1 phenotype while other strains such as *Lactobacillus brevis* G-101 (*L. brevis*), *L. acidophilus* LA1 and *L. plantarum* CLP-0611 trigger M2 macrophages to exert the anti-inflammatory function thereby initiate macrophage polarization from pro-inflammatory M1 phenotype to anti-inflammatory M2 phenotype. *brevis* G-101 and *L. plantarum* CLP-0611 enhance Nrf2 signaling through the production of antioxidants that reduce oxidative stress, and *L. acidophilus* LA1 inhibits endoplasmic reticulum (ER) stress in macrophages, reducing IL-12 and TNF-α expression (94–97). Butyrate also targets the receptor GPR109A on macrophages, which blocks NLRP3 inflammasome activation and increases IL-10 release (98, 99). These signals mutually interact to reshuffle the inflammatory microenvironment of autoimmune diseases. In a study conducted by Feng Li et al. the adoptive transfer of M2 instead of M1 macrophages had a considerable impact of decreasing the intensity of SLE in clodronate-treated mice and Activated Lymphocyte-Derived DNA (ALD-DNA) (100).

*Lactobacillus delbrueckii* (*L. delbrueckii*) and *L. rhamnosus* not only induce an anti-inflammatory phenotype in M2 macrophages but also make M1 macrophages convert to a less inflammatory phenotype by suppressing the immune activation markers like CD14, CD80, and HLA-DR also decrease pro-inflammatory cytokines like IL-12, IL-1β, and TNF-α and increase anti-inflammatory cytokines like IL-10 and TGF-β in SLE patient macrophages (**Figure 2**) (101). SCFAs like butyrate, which are generated through probiotic fermentation of dietary fiber, inhibit HDACs, leading to epigenetic silencing of pro-inflammatory cytokines (e.g., IL-1β, TNF-α) by downregulating NF-κB and upregulating IL-10 by activating STAT3 (98, 102, 103). Thus, probiotics may be considered as a therapeutic agent in SLE by regulating the macrophage to an anti-inflammatory phenotype that would promote disease conditions (101).

Probiotics such as *L.rhamnosus* and *L. delbrueckii* modulate DCs via the aryl hydrocarbon receptor (AhR) by tryptophan metabolites, inducing indoleamine 2,3-dioxygenase (IDO) for Treg differentiation and suppress autoreactive T-cells (104–106) (**Table 2**). *L. delbrueckii* and *L. rhamnosus* secrete EPS that act on TLR2, activating the PI3K/Akt/mTOR pathway to reduce DC surface markers (CD80/CD86) and induce IL-10 production, inducing tolerogenic DCs (107–109). In SLE, certain probiotics are found to impede DC migration by downregulating of CCR7 and CXCR4 chemokine receptors through AhR and NF-κB pathway mechanisms thereby reducing their homing to lymph nodes and limiting autoreactive T-cells (108). Probiotics also modulate the activity of DCs and provoke anti-inflammatory functions, including the induction of IL-10 production, thus ensuring immune homeostasis in SLE (**Figure 2**) (110, 111).

Probiotics enhance natural killer (NK) cell activity in SLE through metabolites such as SCFAs and EPS. *L. casei* (*Lactobacillus casei*) produces acetate, a SCFA that activates the NKG2D receptor pathway on NK cells, boosting granzyme B and perforin release to eliminate autoreactive B and T cells (112, 113). EPS of *Lactobacillus* species engage with TLR2, stimulating the PI3K/Akt/mTOR pathway to enhance IFN-γ production and restore NK cell cytotoxicity suppressed in SLE (**Figure 2**) (113). Probiotic metabolites, such as SCFAs, can enhance NK cell function through epigenetic mechanisms like inhibition of histone deacetylase (HDAC) (112). Concurrently, probiotics suppress IL-6/STAT3 signaling in dendritic cells, activating NK cells for specific elimination of autoreactive lymphocytes and reducing systemic inflammation (113). This dual modulation of pathways and metabolites restores NK cell-mediated immune balance in SLE.

#### 3.4.2 Probiotic impact on adaptive immune response

Dendritic cells are of very significant importance in the immune reaction of infected tissue since they can capture different antigens and travel to the lymph nodes, where they expose antigens to naïve CD4+ T cells. This interaction is important for driving T cell polarization towards particular subsets, such as the T helper cells, which then further differentiate into Th1, Th2, Th17, or regulatory T cells (Tregs) (114). In SLE, the generation and function of various subsets of T helper cells specifically Th1, Th2, Tregs, and Th17 cells are greatly compromised, which is the basis of the disease’s complex immunopathology (115–117).

Th1 cells, which are usually implicated in the induction of cell-mediated immunity, their differentiation is induced by T-box expressed in T cells (T-bet) transcription factor. Th1 cells, frequently show increased activity in lupus. This augmented height can lead to the production of pro-inflammatory cytokines such as IFN-γ, which promotes B cell class switching and initiates pathogenic autoantibodies production such as Immunoglobulin G (IgG) antibodies, most notably anti-dsDNA antibodies, typical lupus-characteristic markers, that induce additional inflammation and tissue damage (115, 118). Probiotics can modulate immune functions through the modulation of various T cell subsets, for instance, Th1 cells. Some probiotics have been reported to modulate T-bet expression. In Shi et al, *Lactobacillus rhamnosus* GG (LGG) was shown to modulate T-bet expression and Th1 differentiation via two metabolite-mediated pathways regulation of T-bet, EPS engage the TLR2/MyD88 dendritic cell pathway with subsequent production of IL-12 and resultant STAT4 phosphorylation that directly amplifies T-bet, and SCFAs, such as butyrate, repress histone deacetylases (HDACs), enhancing *Tbx21* promoter acetylation to increase T-bet expression. a critical transcription factor that regulates Th1 polarization and IFN-γ production. The study finds that LGG can be employed to regulate immune homeostasis by regulating the Th1/Th2 ratio, which may reduce overactivated inflammatory reactions involved in autoimmune disorders such as SLE (119).

In their characterization of a pristane-induced *BALB/c* mice model of SLE, Mardani et al. demonstrated that treatment with the probiotic microbes *L. casei* and *Lactobacillus reuteri* (*L. reuteri*) significantly lowered Th1-mediated inflammation accompanied by reduced levels of IFN-γ and a notable reduction in serum anti-dsDNA autoantibody titers, which are one of the hallmark indicators for lupus severity (**Figure 2**) (120).

Th17 cells are also regulated by master transcription factors like retinoid-related orphan receptor gamma t (RORγt) and signal transducer and activator of transcription 3 (STAT3). STAT3 itself governs expression of RORγt and IL-17A, whose expression is a must for differentiation as well as Th17 cell functions (121). They predominantly secrete IL-17A, a pro-inflammatory cytokine. The excess of Th17 cells in SLE patients exacerbate immune dysregulation, causing severity and progression of the disease (117). It has been demonstrated that Th17 cells promote the development of SLE (122). Elevated IL-6 levels in SLE patients correlate with Th17 cell expansion and disease activity (123). Several probiotic strains have been shown to modulate Th17 cells and their related cytokines in lupus models through specific metabolites and signaling pathways metabolites and signaling pathways. *L. acidophilus* produces SCFAs like acetate, which activate Intercellular adhesion molecule-3-Grabbing Non-integrin Receptor 3 (SIGNR3) receptor on dendritic cells, suppressing IL-6/STAT3 and RORγt pathways to inhibit Th17 differentiation while promoting Treg expansion, helping restore immune balance (37). *L.plantarum* and *A.muciniphila* secrete metabolites that bind TLR2, downregulating NF-κB to block the IL-23/Th17 axis, Additionally, A. muciniphila enhances gut-derived tryptophan metabolites that activate the AhR, suppressing RORγt and elevating IL-10 (**Figure 2**) (77). In pristane-induced lupus, *Lactobacillus* strains reduce Th17 frequency by inhibiting IL-1β/NLRP3 inflammasome signaling and upregulating TGF-β/Smad3 to expand Tregs (120). Additionally, Lactobacillus administration in NZB/W F1 mice enhances Nrf2-mediated antioxidant responses, mitigating oxidative stress that drives Th17 inflammation (124, 125). These metabolite-pathway interactions collectively restore immune balance in lupus models (**Table 2**).

Th2 differentiation is triggered by cytokines such as IL-4 and the transcription factor GATA Binding Protein 3 (GATA3). TH2-released IL-4, IL-5, and IL-13 to a great extent causing B cell proliferation, class switching predominantly to IgG1 and IgE, and recruitment of eosinophils (126, 127). Th2 cells, in SLE, promote disease initiation by causing autoantibody formation which lead to immune complex deposition and chronic inflammation induction. Excess IL-4 and IL-13 induce IgE class switching, which has been associated with the severity of lupus (116). Probiotics modulate Th2 responses and B cell activity in SLE through metabolites such as bacterial DNA, SCFAs, and EPS. *L. rhamnosus* DNA activates TLR9 on intestinal epithelial cells, triggering the IRF3/STAT1 pathway to upregulate PD-L1 expression, which induces apoptosis in hyperactive Th2 cells via PD-1 binding (128). Based on evidence reviewed in Cristofori et al, SCFAs like butyrate enhance TGF-β/Smad3 signaling in B cells, promoting IgA class switching while suppressing IgE production through HDAC inhibition and GATA3 downregulation (129). Concurrent evidence from a systematic review suggests that Lactobacillus-derived EPS bind TLR2, inhibiting IL-4/STAT6 signaling to reduce Th2 polarization and autoantibody generation (**Figure 2**) (51). In SLE, these mechanisms restore immune balance by curbing pathogenic Th2 overactivation (e.g., IL-5/IL-13) while enhancing regulatory IgA synthesis and Treg-mediated suppression of autoreactive B cells (51, 129). Further studies are needed to delineate strain-specific effects on SLE-associated Th2 dysregulation.

Treg cells are essential for preserving immunological tolerance and averting autoimmune disorders like SLE. They are primarily regulated by the transcription factor forkhead box P3 (*FOXP3*), which is essential for their development, function and their suppressive activity, any mutations in the *FOXP3* gene can impair the Tregs activity, leading to autoimmune disorders. Treg function by suppressing the activation of effector T cells, including Th17 cells, and preventing excessive inflammatory responses. In SLE, the imbalance between Tregs and effector T cells, results in a loss of immune tolerance and contributes to the pathogenesis of the disease. In particular, low Treg numbers or Treg dysfunction aggravates autoimmune activity and results in SLE development. In addition, Tregs in SLE patients are usually of weaker regulatory function towards inflammation, and medications with the capacity to enhance Treg function can restore immune homeostasis in these individuals (130–133). Probiotics such as *L. delbrueckii*, *L. rhamnosus*, *L. reuteri*, and *L. acidophilus* induce CD4+CD25+Foxp3+ Tregs and suppress Th17 cells in lupus models via metabolite-mediated signaling pathways (37, 125, 134). *L. acidophilus* produces SCFAs such as acetate, which activates the SIGNR3 receptor, suppressing IL-6/STAT3 and RORγt pathways to inhibit Th17 differentiation while inducing Foxp3, IL-10, and TGF-β to stabilize Tregs (**Figure 2**) (37). *L. delbrueckii* and *L. rhamnosus* increase regulatory T cell differentiation and regulatory potential in pristane-induced lupus mice, associated with enhanced FOXP3 expression and reduced inflammation (134). While EPS from *L. delbrueckii* have been shown to engage TLR2 and activate PI3K/Akt signaling pathways in immune cells (135). The direct involvement of EPS-TLR2 binding and PI3K/Akt/mTOR activation in Treg amplification in lupus models remains to be fully demonstrated. Probiotics more broadly impact immune regulation through modulation of the PI3K/Akt pathway, which supports their potential role in enhancing regulatory T cell functions (136). *L. reuteri* produces tryptophan metabolites, including indole derivatives, that activate AhR, leading to Foxp3 expression and IL-10 production to suppress Th17 cells (104, 137, 138). *L. casei* and *L. reuteri* also enhance antioxidant enzyme activities such as glutathione peroxidase, suppressing oxidative stress and stabilizing regulatory T cells by preventing ROS-dependent mTOR activation, which plays a role in immune regulation in lupus models (124). Synergistically, *L. acidophilus* augments the activity of tacrolimus by promoting the proliferation of SIGNR3-associated Treg and inhibiting Th17, reestablishing the balance of Th17/Treg ratios in lymphoid tissues (37). All these combined restore immune homeostasis in lupus by Treg predominance and inhibiting pathogenic Th17 activity.

B cells are equally significant components of the immune system whose formation and function are regulated by major transcription factors such as Paired Box Protein 5 (PAX5), Early B-cell Factor 1 (EBF1), E2A Transcription Factor (E2A), and Interferon Regulatory Factor 4 (IRF4). These proteins play critical functions in commitment, differentiation, and activation of B cell lineage (139). In SLE, B cells contribute to pathogenesis through to the generation of autoantibodies. B-cell Activating Factor (BAFF) and A Proliferation-Inducing Ligand (APRIL) are critical cytokines responsible for B-cell survival and differentiation, and their overexpression in SLE supports proliferation of autoreactive B cells (140, 141). The increased B cell differentiation into plasma cells allows for autoantibody production leading to immune complex deposition in tissues causing inflammation and tissue injury (140). Probiotics have been investigated as a complementary treatment for SLE, hopefully affecting the immune system by modulating the activity of B cells and reducing inflammation. In SLE, the action of probiotics on BAFF and APRIL can be complex. Conversely, Intestinal epithelial cells can generate APRIL and BAFF in response to some probiotics or their metabolites, which causes B-cell class flipping to IgA-secreting plasma cells (**Figure 2**) (139, 142). This is beneficial for mucosal immunity, as IgA acts as a first line of defense in the gut, preventing pathogen adhesion and modulating mucosal immune reactions (129). Probiotics such as *Lactobacillus* and *Streptococcus thermophilus* (*S. thermophilus*) are observed to induce mucosal IgA production by SCFAs such as butyrate, which inhibit HDACs to epigenetically activate TGF-β/Smad3 signaling in DCs, resulting in class-switching of B cells to IgA+ plasma cells in the gut mucosal lining in a dose-dependent manner (143, 144). *L. rhamnosus* and *L. delbrueckii* produce TLR2-stimulating EPS, which activate the PI3K/Akt/mTOR pathway in DCs to induce IL-10 and retinoic acid production, enhancing IgA synthesis further and producing tolerogenic DCs that suppress autoreactive B cells (107, 108). However, an overproduction of probiotic metabolites like peptidoglycan fragments or bacterial DNA can hyperactivate DCs via TLR2/9 or NOD2, boosting BAFF/APRIL production via NF-κB/MAPK pathways, potentially triggering systemic autoreactive B cells and exacerbating SLE autoimmunity (139–141). Lactobacillus tryptophan metabolites (such as indole-3-lactic acid) mitigate this danger by activating the AhR to suppress BAFF/APRIL overproduction via mTOR, restoring systemic tolerance and mucosal immunity (108, 144). Thus, probiotics’ dual effects enhancing IgA while modulating DC-B cell crosstalk require strain and dose specific optimization in SLE.

#### 3.4.3 Signaling pathways influenced by probiotics

Cytokines play a critical role in SLE immune regulation where patients primarily exhibit an upregulation of proinflammatory cytokines (e.g., TNF-α, IL-6, IL-17A, IFN-γ) and downregulation of anti-inflammatory cytokines like IL-10 and TGF-β, that play a role in increased autoimmunity expressed by SLE (145). Probiotics overall effect on cytokines in SLE is by reducing the proinflammatory cytokines and enhancing the anti-inflammatory cytokines. To suppress pro-inflammatory cytokines *L.delbrueckii* and *L.rhamnosus* inhibit NF-κB and MAPK signaling pathways, reducing levels of TNF-α, IL-6, and IFN-γ production (146, 147). Butyrate, a SCFA derived from probiotic metabolism, downregulates the transcription factor RORγt in Th17 cells, curtailing IL-17A secretion a key driver of lupus nephritis considerably lowering pro-inflammatory cytokines like TNF-α and IL-6 (146) (**Table 2**). Concurrently, SCFAs and EPS from probiotics synergistically activate dendritic cells and macrophages to secrete IL-10 and TGF-β, promoting Treg expansion and suppressing autoreactive lymphocytes (98, 101). *L. plantarum* enhances AhR activation, elevating IL-10 and restoring gut barrier integrity, which further stabilizes immune homeostasis (77, 113).

NF-KB hyperactivation in SLE increase inflammation by enhancing autoreactive B cell survival and proinflammatory cytokine production (148). Probiotics like *L. rhamnosus* LC-STH-13 inhibit NF-κB activation by blocking TLR9 signaling and preventing IκBα degradation, thus reducing pro-inflammatory cytokines such as TNF-α, IL-6, and IL-1β in lupus-prone mice (**Figure 2**) (147). SCFAs produced by probiotic fermentation, bind to G protein-coupled receptors GPR41 and GPR43 on immune cells, attenuating NF-κB and MAPK signaling pathways in neutrophils and suppressing oxidative burst and cytokine release (146, 149). This dual inhibition attenuates systemic inflammation and tissue damage in SLE models (51).

Enhanced HDAC activity in SLE worsens immune dysregulation through the repression of anti-inflammatory genes such as Foxp3 and promotion of Th17 responses (150, 151). Probiotics counteract this effect by generating SCFAs such as butyrate and propionate, which are HDAC inhibitors. The SCFAs enhance histone acetylation within the Foxp3 locus, promoting Treg differentiation and preventing Th17 cell development (152). Concurrently, in new research, lactate-mediated histone lactylation, and notably H3K18la, have been recognized as a novel epigenetic process linking cellular metabolism with immunological memory (153–155). SCFAs also contribute to chromatin remodeling by stabilizing accessibility at anti-inflammatory gene regions (156). Together, these epigenetic modifications help restore immune tolerance and may reduce SLE flares (152).

Probiotic-derived EPS activate TLR2 on DCs, fostering a tolerogenic phenotype characterized by IL-10 secretion and reduced autoreactive B/T cell activation (108, 146). Additionally, *L. casei* increases nicotinamide levels, which inhibit reactive oxygen species (ROS) production in neutrophils, preventing NETosis a process implicated in systemic lupus erythematosus pathogenesis. Lactobacillus casei combined with inulin improves antioxidant enzyme activities in humans, reducing oxidative stress (157). Nicotinamide also upregulates NAD+ metabolism, supporting mitochondrial health in regulatory T cells and enhancing their immunosuppressive capacity (125).

## 4 Nutritional synergy: the role of ginger and vitamin D

Nutrition and supplements significantly affect control of immune response and microbiota homeostasis, with implications for treatment of autoimmune diseases, like SLE. Gut microbiota has also increasingly been recognized as powerful immunomodulatory reagents in regulating inflammation and autoimmune diseases. Food constituents, such as some of the bioactive molecules within food, can directly affect gut health by controlling microbial diversity and improving beneficial probiotic function. Nutritional supplements, such as vitamins and minerals, also contribute to immune control through cytokine production modulation and cell signaling pathways. Optimization of the composition and functionality of the gut microbiota by diet and supplementation in SLE could present an alternative therapeutic strategy to conventional management. The role of nutritional interventions (e.g., ginger) and supplementations (e.g., vitamin D) in modulating SLE symptoms, probiotics, and gut integrity is discussed in this section.

### 4.1 Ginger: a bridge between food and medicine

Food and medicine have a long history in the natural world, and both share the ability to make a strong influence on health and well-being. Many civilizations have known over the centuries that certain foods, especially herbs and spices, have therapeutic properties as well as nutritional worth. This holistic viewpoint emphasizes how our food choices have a direct effect on our mental and physical well-being (161). Among various dietary components known for their health benefits, ginger stands out as a representative dietary intervention that exerts significant immunomodulatory and anti-inflammatory effects (162). Its bioactive compounds, particularly 6-gingerol, have been shown to impact gut microbiota and enhance the activity of probiotics, thereby improving the regulation of immune responses in SLE.

6-gingerol represents one of the major bioactive ingredients of fresh ginger, being one of the most relevant supplements related to nutrient factors with possible activities of modulation towards immune functions. It possesses well-documented anti-inflammatory and antioxidant properties (163). This compound has lately appeared to be significantly involved also in the enhancement of gut health, especially related to probiotics (164).

The primary way in which 6-gingerol mediates its functions is by promoting probiotic adhesion to intestinal epithelial cells. Research has demonstrated that6-gingerol improves probiotics’ adherence to colonic epithelial cells, particularly those of *L. acidophilus* and *Bifidobacterium bifidum* (*B. bifidum*). in a dose-dependent manner (164). This improved adhesion is crucial because it fosters the colonization of beneficial bacteria, which is necessary for maintaining gut homeostasis and supporting immune modulation which may be beneficial in alleviating conditions such as SLE (164).

Further evidence shows that 6-gingerol can alter gut bacteria diversity and composition to benefit those with inflammatory and immune-related diseases. For instance, 6-gingerol increased the proportion of the phylum Bacteroidetes in high-fat diet-induced obese mice, therefore decreasing the F/B ratio generally related to obesity and inflammation (165).

Based on research by Ali et al, 6-gingerol decreases the development of NETs significantly in lupus models, therefore being protective against the cardinal pathological features associated with lupus-inflammation and autoantibody formation. Furthermore, the study elaborates on the antioxidant properties of 6-gingerol by noting its potential to reduce levels of ROS and recover glutathione to protect cells against oxidative stress and inflammation (162). This effect may be further enhanced in a synergistic manner by probiotics through the reduction of NETs while exhibiting antioxidative properties that lower ROS production and cellular cytotoxicity (92). In another study, 6-gingerol has been found to lower autoantibody levels in a lupus mouse model. The cell-free DNA and myeloperoxidase-DNA (MPO-DNA) complex levels were significantly decreased after treatment, indicating a notable decrease in plasma NET levels. Significant decreases were also noted in primary autoantibodies such as total IgG, anti-β2 glycoprotein I (anti-β2GPI), and anti-dsDNA (162). The findings highlight the synergistic effect of probiotics, which can regulate the amount of autoantibodies along with the 6-gingerol (51).

Moreover, some publications have identified a reduction of inflammatory cytokine levels in SLE patients that might be diminished with the intake of probiotics, highlighting their potential immunomodulatory effects (166). In parallel, an experimental treatment of 6-gingerol on mice induced with lupus showed reductions in the levels of the pro-inflammatory TNF-α and IFN-γ isotypes, pointing to its good effects on SLE’s usual general inflammation aspects (162). These findings suggest that a combined therapeutic strategy involving both probiotics and 6-gingerol may exert complementary or synergistic effects by targeting overlapping inflammatory pathways, thereby offering enhanced clinical benefit in managing SLE.

In addition to their ability to balance cytokine production, probiotics enhance the activity of Treg cells (125). Perhaps 6-gingerol may work in collaboration with probiotics by additionally enhancing Treg proliferation and activity, hence autoimmune flares and symptoms and enhancing SLE symptoms (167). Interestingly, 6-gingerol also was found to suppress the NF-κB pathway, thereby lessening the inflammatory cytokine output (168). This increases the effectiveness of probiotics in inhibiting this route, which may provide a promising treatment for autoimmune conditions like SLE (147).

In summary, the synergistic effect of 6-gingerol in combination with probiotics offers a potential direction of study in managing SLE. Through their collective capacity for modulating the immune system and lessening inflammation, these therapies hold the potential to be utilized as adjunctive therapies in SLE management.

### 4.2 Vitamin D: a vital nutrient in health and autoimmunity

While nutritional components like ginger regulate gut health and immunity through the natural consumption of foods, nutritional supplements like vitamin D are major regulators of immune activity and balance of gut microbiota. Vitamin D deficiency is observed in SLE patients and also reported to be linked with disease activity. Vitamin D has an important function in inhibiting inflammation and enhancing immune tolerance in SLE by functioning in collaboration with probiotics and immune cells. Many studies have reported that large percentages of SLE patients have low levels of vitamin D, and in some studies, as much as 73.3% insufficiency and 23.3% frank deficiency (169). The effects of corticosteroid medication, increased sunscreen use, and decreased sun exposure are also contributing reasons for this insufficiency (170). It is mostly observed that higher disease activity scores are equivalent to low levels of vitamin D, and therefore low levels can be implicated in worsening symptoms and overall poor health outcomes in these patients (169). Indeed, vitamin D levels were reported to inversely relate to the SLE Disease Activity Index, in which low levels of vitamin D were indicative of greater disease severity (169). Deficiency in vitamin D may also worsen the systemic symptoms, such as fatigue, that are experienced by most SLE patients (171).

In recent studies, consideration has been given to an interaction existing between vitamin D and gut microbiota. Vitamin D particularly modulates the intestinal microbiome by improving the growth of bacteria, usually regarded as beneficial or through enhancement of antimicrobial properties of gut epithelium. Vitamin D may also act through VDR to lower microbial dysbiosis and improve intestinal barrier function with a rise in SCFA production by commensal bacteria. These changes not only improve gut health but also enhance immune function by regulating intestinal inflammation (172–174). 1,25-dihydroxyvitamin D_3_ (1,25-(OH)_2_-VitD_3_) is the active form of vitamin D_3_, which is known for its immunomodulatory effect. Research showed that it can interact with the VDR receptor, enhancing its action. VDR is present on several immune cells’ surfaces, including B lymphocytes, macrophages, T cells, and dendritic cells enhancing the immune modulatory effect (175).

In SLE, there is a general overactivation of T helper cells, with the Th17 cells especially contributing much to inflammation. Indeed, active vitamin D was shown to suppress Th17 cell differentiation and activity, hence diminishing their contribution to the inflammatory response characteristic of SLE. In contrast, 1,25-(OH)2-VitD3 enhanced Tregs’ T cell differentiation-required for maintenance of immune tolerance and resistance to autoimmunity (176, 177). probiotics also restrict production of Th17, yet concurrently it promotes proliferation of Tregs (120, 125). This might provide insight into the different aspects that demonstrate potential synergic actions of probiotics with vitamin D supplementation on each.

Other studies cite the ability of 1,25-(OH)2-VitD3 to reduce autoantibodies such as anti-dsDNA by acting negatively on B cells through suppression of its growth and apoptosis induction of the autoreactive B cells (178). Long-term observational data further correlate Vitamin D sufficiency with reduced autoantibody titers such as anti-dsDNA and disease activity (179). 1,25(OH)2D3 has been shown to suppress mitogen-stimulated IgG production in peripheral blood mononuclear cells from both healthy individuals and inactive SLE patients, indicating a direct inhibitory effect on IgG synthesis (180). Long-term observational data further correlate Vitamin D sufficiency with reduced autoantibody titers and disease activity. probiotics have been shown to lower the serum levels of Antinuclear Antibodies (ANA) (51). This activity cites a hopeful role for 1,25-(OH)2-VitD3 and probiotics as a therapy for autoimmune diseases.

Furthermore, vitamin D also showed significant inhibition of NF-κB pathways (181), and a regulatory effect regarding cytokine production, thus it inhibits proinflammatory cytokines in SLE pathogenesis, including IFN-γ, IL-17, IL-23, IL-6, and TNF-α (182–184), while stimulating anti-inflammatory cytokine production like IL-10 and TGF-β (185, 186). Similarly to probiotics, which are also able to enhance anti-inflammatory cytokines and decrease pro-inflammatory cytokines, probiotics are also believed to block the NF-κB pathway, showing possibilities for a synergistic approach with vitamin D to further reduce inflammation in SLE (51, 147, 166).

Probiotics exert anti-inflammatory and immunomodulating effects and can therefore also act synergistically with vitamin D. Probiotics can enhance vitamin D receptor (VDR) protein expression, improving active vitamin D absorption, which acts in turn to potentiate immune response regulation (187). Research has demonstrated that probiotics such as LGG *and L. plantarum* enhanced the expression of VDR protein and *L. plantarum* alone increased VDR transcriptional activity in Human Colorectal Carcinoma Cell Line 116 (HCT116) cells. Furthermore, while probiotics had no effect on VDR (-/-) mice, they were demonstrated to provide physiological and histologic protection against Salmonella-induced colitis in VDR (+/+) animals (187). Recent evidence suggests that vitamin D-probiotics co-supplementation might increase VDR expression and improve the anti-inflammatory action of these compounds (188). Co-supplementation is a novel approach for the therapy of SLE and other autoimmune disorders. Through the utilization of vitamin D’s immune-modulatory effect and the ability of probiotics to enhance VDR signaling and gastrointestinal health, this treatment may deliver synergistic benefits over current treatments.

In conclusion, Vitamin D may have a multirole in immune function regulation, creating anti-inflammatory effects and supporting general health. Its interaction with gut microbiota and synergistic action with probiotics make it a very promising adjunctive therapy for autoimmune diseases, including SLE.

## 5 Translational bottlenecks from the laboratory to the clinic

Probiotic supplementation in patients with systemic lupus erythematosus (SLE) shows promising potential benefits but also requires careful consideration of safety. The long-term effects of prolonged probiotic use are a topic of growing research and debate. While probiotics are generally considered safe for short-term use, extended or indiscriminate consumption may lead to unintended consequences, particularly in specific populations, theoretical risks include systemic infections, immune overactivation, and metabolic disturbances, especially in immunocompromised or severely ill individuals (181).

### 5.1 Long-term effects due to prolonged usage of probiotics

#### 5.1.1 Disruption of gut microbiota balance

Prolonged probiotic use may interfere with the natural recovery of gut microbiota, particularly following antibiotic treatment. A study published in Cell demonstrated that probiotics delayed gut microbial reconstitution post-antibiotics, whereas fecal microbiota transplantation (FMT) or natural recovery restored diversity more effectively (189). In healthy individuals, probiotics may transiently colonize the gut but fail to integrate into the native microbiome, potentially destabilizing microbial diversity over time (189). This destabilization could reduce resilience to environmental perturbations, such as infections or dietary changes, and alter metabolic functions critical for host health (190).

#### 5.1.2 Gut barrier dysfunction

In cases of intestinal permeability generally caused by overwhelming inflammation, surgery, or in inflammatory bowel disease (IBD), probiotics may pass through the compromised gut barrier into the bloodstream and cause systemic infection. For example, immunocompromised patients or those with central venous catheters risk bacteremia linked to probiotic organisms like *L. rhamnosus* GG (191, 192). Such translocation mandates strain selection and patient-specific risk assessment before extended probiotic use (191, 193).

#### 5.1.3 Antibiotic resistance

Certain probiotic species possess antibiotic-resistant genes that can be transferred to pathogenic bacteria via horizontal gene transfer. For instance, mutations in LGG strains have been associated with resistance to rifampin and potential for long-term evolution of the microbial community in response to probiotic pressure (194, 195). Misuse of probiotics may also enhance the presence of antibiotic-resistant forms within the intestinal environment and hence facilitate infection treatment complexities in the clinic (196, 197). Wise probiotic screening for mobile genetic elements and resistance markers must be conducted to minimize this risk (198).

#### 5.1.4 Immune system modulation

While probiotics may enhance immune function in the short term, long-term administration has the potential to overactivated the immune system, worsening chronic inflammation or autoimmune disease (59). Animal research indicates that exposure to antibiotics early in life, frequently combined with probiotics, perturbs immune development and enhances lifelong risk for metabolic diseases such as obesity and diabetes (199). For instance, antibiotic- and probiotic-treated neonatal mice have unbalanced T-cell responses and reduced tolerance to dietary antigens, highlighting the delicate balance required in immune training (199).

#### 5.1.5 Dependency and reduced native microbial resilience

Continuous probiotic supplementation may suppress the growth of native gut bacteria, leading to functional dependency. Studies show that probiotics can inhibit the recovery of beneficial genera like Bifidobacterium and Eubacterium after dysbiosis, delaying the restoration of a healthy microbiome (200). In healthy individuals, long-term use might diminish the gut’s intrinsic ability to self-regulate, reducing colonization resistance against pathogens like Chloridoids difficile or Salmonella (52, 200, 201).

#### 5.1.6 Variable efficacy and individual responses

Probiotic effects are highly strain- and individual-specific. For example, only 1 in 7 randomized controlled trials demonstrate successful gut colonization of administered probiotics, underscoring the variability in efficacy (202). Personalized approaches are critical, as host genetics, diet, and baseline microbiota composition significantly influence outcomes (203, 204). Emerging strategies, such as microbiome profiling and metabolomic analysis, are being explored to tailor probiotic therapies to individual needs, though standardized protocols remain under development (205).

### 5.2 Safety concerns in immunosuppressed patients

Probiotic application in immunosuppressed patients is extremely important due to their compromised immune protection. Systemic infection is among the serious hazards because live probiotic bacteria (e.g., Lactobacillus or Saccharomyces) may translocate across the gut barrier and induce septicemia, fungemia, or endocarditis. While rare, these infections are improperly recorded in immunocompromised individuals, particularly central venous catheter patients or those with severe intestinal permeability, emphasizing the need for strain-specific risk assessments (193, 206). Imbalance of flora is another concern, probiotics unintentionally can trigger overgrowth of commensal or pathogenic bacteria in an uncontrolled gut environment. For example, immunocompromised patients on chemotherapy or antibiotics experience reduced microbial diversity and are susceptible to probiotic-induced disturbances, e.g., overgrowth of Chloridoids difficile or fungal domination (193).

Immune overactivation poses a theoretical yet significant risk. Probiotics modulate dendritic cell function and cytokine release (e.g., IL-6, IFN-γ), possibly exacerbating autoimmune or inflammatory disease in predisposed hosts. BAFF and APRIL pathways are particularly concerning, as overexpression initiated by probiotic metabolites like bacterial DNA or exopolysaccharides has been linked with autoantibody production and lupus-like syndromes (193, 207). Finally, metabolic disturbances, though inadequately reported, represent a theoretical risk. Probiotic strains with urease activity or D-lactate production could theoretically contribute to hyperammonemia or metabolic acidosis in patients with hepatic or renal impairment (193). While evidence is limited, these risks underscore the necessity for rigorous monitoring and personalized probiotic strategies in immunosuppressed populations.

### 5.3 Gender-related differences in probiotic response

Sex-specific probiotic efficacy differences may arise due to the interaction of probiotics with sex hormone signaling, most notably estrogen. *L.reuteri* was seen to improve the secretion and expression of intestinal hormones, including estrogen receptor-beta (ER-β), which can improve anti-inflammatory effects by promoting IL-10 levels and Treg differentiation. These are especially relevant in females, in whom estrogen signaling boosts probiotic-induced immune modulation (208).

Further, animal studies demonstrate sex-dependent differences in response to *L. reuteri* therapy. In mice, *L. reuteri* supplementation increases beneficial bacteria and immune cell populations more significantly in females compared to males, which is probably explained by the immunomodulatory actions of estrogen. This agrees with the observation that probiotic effects may be more potent in females, and sex must be considered as a biological variable when conducting research and therapy with probiotics (209).

Mechanistically, probiotics can interact with SLE pathophysiology in various ways by gender. In females, estrogen dominance enhances gut permeability via claudin-2 upregulation, a process counteracted by probiotics like *L. rhamnosus* GG, which strengthen tight junctions and reduce bacterial translocation (210, 211). Conversely, probiotics that metabolize estrogen (e.g., *L. plantarum*) could lower systemic estrogen levels, indirectly dampening B-cell hyperactivity (77). In men, testosterone’s immunosuppressive effects might synergize with probiotics to suppress autoimmunity, but this is not yet investigated in SLE models (212). Despite these insights, most clinical trials fail to stratify outcomes by sex, obscuring gender-specific mechanisms. Addressing this gap is critical, as personalized probiotic strategies could optimize therapeutic benefits while mitigating risks in SLE management (51).

### 5.4 Dosage, survival, and delivery systems of probiotics

Most investigations of probiotic effectiveness in SLE underscore the importance of optimum dose maximization for immune modification. Optimum concentrations of 10^7 CFU/mL for *L. rhamnosus* and 10^5 CFU/mL for *L. delbrueckii* have been determined from an ex vivo experiment conducted with peripheral blood mononuclear cells (PBMCs) in SLE patients. Both of these probiotics, in those dosages, enhanced regulatory T cell-related gene expression and lowered proinflammatory cytokines as markers of high immunomodulatory activity (146).

Survival of probiotic bacteria through the gastrointestinal tract is crucial to their functionality because they must survive harmful conditions of gastric pH, bile salts, and digestive enzymes to maintain viability and functionality (213, 214). Formulation with protective measures, such as the use of encapsulation techniques using polymeric materials, shields the probiotics from stresses, allowing them to survive and target specifically in the intestine (215, 216). For example, alginate hydrogels find widespread use on the basis of acid insolubility and ability to form protective microcapsules that confer probiotic stability and controlled release (215).

Probiotic delivery systems can be broadly classified into conventional and non-conventional systems. The conventional drug delivery systems include beads, capsules, and tablets, with the oral route being the most used for convenience, economy, minimal risk of infection, and good patient compliance (213, 217). Technological innovation in formulation has witnessed the advent of microencapsulation and nano-encapsulation techniques, where there is precise control over particle size and surface chemistry, further promoting probiotic viability and enabling targeted delivery within the gastrointestinal tract (216). Methods like electrospun alginate nanofibers have been found to provide improved protection for probiotics against gastric conditions and improved survival rates compared to non-encapsulated ones (216).

In addition, other administration routes such as nasal, transdermal, vaginal, and rectal are investigated to potentially enhance therapeutic effects and maximize clinical efficacy of probiotics beyond oral delivery (218). These alternative routes may help overcome some limitations of oral delivery, such as degradation in the GI tract, and offer new opportunities for probiotic therapies.

### 5.5 Dosage and timing of administration of nutritional supplements

In SLE animal models, 6-gingerol, has been administered at about 10 mg/kg intraperitoneally three times per week from week 4 of treatment. For example, in TLR7 agonist-induced lupus mouse model, delayed treatment with 6-gingerol from week 4 and for 2 weeks greatly reduced plasma NET levels, autoantibody formation (e.g., anti-dsDNA and anti-β2GPI antibodies), and thrombosis but not total leukocytes or spleen size (162). Other studies using oral gavage reported doses ranging from 100 to 250 mg/kg daily in mice, which effectively reduced lupus-related inflammation and symptoms without observed toxicity (219, 220). While no direct clinical trials provide data on optimal human dosing of 6-gingerol for SLE, ginger extracts are generally regarded as safe, with human doses up to 2 grams daily (approximately 25 mg/kg for a 70 kg adult) showing good tolerability in other contexts (219). Translating findings from mice to humans would require careful dose scaling and clinical validation to establish safety and efficacy in SLE patients.

For vitamin D supplementation in SLE patients with deficiency (serum 25-hydroxyvitamin D < 20 ng/mL), clinical practice guidelines are typically vitamin D3–8000 IU daily for 8 weeks and then maintenance on 2000 IU daily. For vitamin D insufficiency (21–29 ng/mL), the dosing is 8000 IU daily for 4 weeks followed by an identical maintenance regimen (221). Importantly, this dosing strategy has been found safe, with no reported incidents of hypercalcemia (221, 222). The benefits include improved SLEDAI scores and reductions in inflammatory markers, supporting the necessity of long-term vitamin D supplementation, ideally lasting at least 6 to 12 months, to achieve maximal therapeutic benefits in SLE patients (221–223).

### 5.6 Limitations of direct administration and the rationale for delivery systems

In moving from laboratory findings to clinical applications, one critical bottleneck is the limited effectiveness of direct administration of therapeutic agents such as probiotics, 6-gingerol, and vitamin D. Probiotics often face harsh gastric conditions, including low pH and bile salts, which drastically reduce their viability before they reach the intestine—the primary site of action (213, 214). Similarly, 6-gingerol suffers from poor water solubility, instability in physiological fluids, and rapid metabolism, all contributing to low oral bioavailability (224). Vitamin D, being lipophilic, is vulnerable to degradation and exhibits variable absorption depending on fat intake and individual metabolic status (225).

These limitations highlight the need for advanced delivery systems such as nano-encapsulation, nano-emulsions, or self-microemulsifying drug delivery systems (SMEDDS) that can protect these compounds from degradation (226–228), enhance mucosal absorption, and enable targeted delivery. Such strategies are essential to overcome pharmacokinetic barriers, improve therapeutic consistency, and enhance patient outcomes in SLE treatment.

### 5.7 The role of microbiota profiling in guiding personalized therapy

Microbiota profiling is a useful path to the personalization of treatment strategies for SLE patients. 16S rRNA gene sequencing and metagenomics are some of the high-throughput sequencing technologies with the ability to identify patient-specific dysbiosis signatures. For example, increased *Lachnospiraceae* or reduced Lactobacillus species can guide the selection of specific probiotic strains (229, 230). Furthermore, unsupervised gut microbiota-based clustering of SLE patients recognized patient subgroups that associated with immune phenotypes and disease activity, which justified the use of microbiota patterns to predict response to therapy (229). Moreover, associating microbiota patterns with cytokine levels, autoantibody profiles, and clinical phenotypes can forecast response to therapies like 6-gingerol or vitamin D (231, 232). This precision medicine approach potentially enables clinicians to individualize treatment regimens through the selection of the best strain, dose, and combination therapies to maximize efficacy and minimize toxicity.

### 5.8 Limited pharmacokinetics and real-world evidence of combination therapy

Pharmacokinetic (PK) and pharmacodynamic (PD) studies are needed to determine how combination treatments such as probiotics, vitamin D, and 6-gingerol interact together in the body to impact both efficacy and safety for treating SLE (233). Currently, there is a significant gap in such studies for these combinations, which hinders the optimization of dosing regimens, prediction of drug interactions, and understanding synergistic or antagonist effects of the agents. As SLE’s immune dysregulation is so complex and with the very real possibility of herb-drug and supplement-drug interactions, PK/PD studies are imperative (233). These studies help to determine the absorption, distribution, metabolism, and excretion profiles of combination agents, characterize their immunomodulatory effects and kinetics in terms of drug concentrations, identify any undesirable effects or toxicities arising from combination use, and inform personalized treatment approaches according to patient-specific factors and disease activity. Without this vital pharmacological data, clinical use of these combinations is largely empirical and could pose safety risks (233, 234).

Effective real-world monitoring of disease activity, flare, and relapse in SLE is at the core of disease management and therapy adjustment, including adjustment of new combination therapies. Modern clinical practice is aimed at frequent measurement of disease activity using validated measures like the SLE Disease Activity Index (SLEDAI) or the British Isles Lupus Assessment Group (BILAG) scores. These are adjusted based on disease severity and patient status (235, 236). Biomarker monitoring is likewise a cornerstone of disease management, including measurement of complement levels (C3, C4), anti-double stranded DNA antibodies, erythrocyte sedimentation rate (ESR), and IFN-α levels (236–239). Sensitive tests like digital ELISA have been found to be helpful in identifying low-level disease activity and even in pre-dicting imminent flares (240). Routine laboratory studies, including complete blood counts, urinalysis, and metabolic panels, are recommended every 3 to 6 months or more frequently during active disease to screen for organ involvement or complications (237, 241). Physicians individualize the frequency of visits based on disease activity, ranging from frequent visits in the context of active lupus nephritis to less frequent follow-up in stable disease, enabling individualized care (241). Emerging biomarkers such as IFNα levels may further improve flare prediction and help guide therapy adjustments, potentially enhancing the management of complex combination therapies (237, 238, 240).

The implementation of sensitive biomarker tests and accredited clinical indices in routine practice is pivotal towards accurate disease monitoring as well as effective therapeutic management. However, an evident lack of real-world evidence in terms of how such monitoring procedures can be applied specifically for combination probiotic, vitamin D, and 6-gingerol therapy in SLE does exist. This shortfall highlights the necessity for future observational registries and studies to evaluate the safety, efficacy, and optimal monitoring practices in these novel combination therapies.

## 6 Challenges and perspectives

Building upon the translational challenges discussed above, future research should now focus on resolving these gaps through innovative solutions and clinical refinement. Several critical areas need targeted investigation to improve the feasibility and effectiveness of probiotic-nutritional interventions in SLE.

1) Formulation Development and Delivery Systems: There are no uniform probiotic and nutritional formulations available for use in the treatment of SLE. Scientific studies need to be conducted for formulation development of optimized delivery systems such as nanoemulsions that would maximize bioavailability, ensure targeted delivery, and stabilize ginger compounds, vitamin D, and probiotics.

2) Limited Mechanistic Studies in SLE Models: While probiotics have been widely studied in terms of their general immunomodulatory activity, few studies have been specifically aimed at their mechanisms in SLE. Further research should investigate how they influence key immune factors such as NK cells, neutrophils, goblet cell-stimulated mucin production, and histone deacetylase regulation in lupus models. Also, additional research on the isolated effect of 1,25-Dihydroxyvitamin D3 on autoantibodies production such as IgA, IgE, IgG and the synergistic effect of probiotics, vitamin D, and ginger bioactive on SLE disease development must be carried out.

3) Personalized Treatments and Sex-Specific Responses: Due to the large disparity in SLE incidence and severity between women and men, individualized probiotic interventions must be investigated. Future studies should identify how treatment responses differ according to sex, genetic susceptibility, microbiota, and individual immune profiles.

4) Comparative Efficacy Studies: Probiotic formulations should be compared against conventional SLE treatments to evaluate their relative effectiveness. This would mean comparing their impacts on SLE-specific immunological biomarkers such as autoantibodies titers, complement proteins (C3, C4), cytokines such as IL-6, IL-10, and TNF-α, and T cell subsets (especially the Th17/Treg ratio), to assess efficacy and monitor disease progression, and clinical manifestations. Clinical trials need to ascertain whether probiotic and nutritional interventions can augment or even replace conventional immunosuppressive medications in select groups of patients.

5) Comprehensive Clinical and Preclinical Studies: Further clinical trials with more rigorous nature are required to establish the effectiveness and safety of probiotic-nutritional interventions for SLE.

By addressing these gaps in research and streamlining therapeutic regimens, probiotics and nutritional synergy can emerge as valuable adjuncts or alternatives to conventional SLE treatments, offering a holistic, personalized, and less toxic approach to disease management.

## 7 Conclusion

Systemic lupus erythematosus is a multicausal autoimmune disorder with pathogenesis strongly associated with intestinal flora dysbiosis, immune imbalance, and metabolic dysregulation. In this paper, the contributions of gut flora, probiotics, and nutritional therapy (e.g., ginger and vitamin D) in SLE are discussed in detail. The gut flora-immunity-metabolism axis is a new target for the treatment of SLE. The simultaneous application of probiotics and dietary interventions is likely to be a safe and effective adjuvant therapy but its use in the clinic has to address issues of formulation standardization, patient suitability, and long-term safety. Integration of basic research and clinical practice will result in more accurate and more comprehensive treatment protocols for SLE patients.

## Glossary

1,25-(OH)₂-VitD₃: 1,25-dihydroxyvitamin D₃ or calcitriol
APRIL: A Proliferation-Inducing Ligand
ALD-DNA: Activated Lymphocyte-Derived DNA
anti-β2GPI: Anti-β2 glycoprotein I
anti-dsDNA: Anti-double-stranded DNA
Anti-IgE: Antibodies that target IgE
anti-aPL: Anti-phospholipid antibodies
anti-RNP: Anti-ribonucleoprotein antibodies
ANA: Antinuclear Antibodies
AHR: Aryl hydrocarbon receptor
(BALB/c) mice: Bagg Albino Laboratory-Bred/c mice
BAFF: B-cell Activating Factor
BILAG: British Isles Lupus Assessment Group
CPSs: Capsular polysaccharides
CD14: Cluster of Differentiation 14
CD25: Cluster of Differentiation 25
CD4: Cluster of Differentiation 4
CD80: Cluster of Differentiation 80
DC: Dendritic Cells
SLEDAI: Disease Activity Index
EBF1: Early B-cell Factor 1
E2A: E2A Transcription Factor
ESR: Erythrocyte sedimentation rate
ER-β: Estrogen receptor-beta
ECPS: Extracellular polysaccharides
EVs: extracellular vesicles
F/B ratio: Firmicutes/Bacteroidetes ratio
Foxp3: Forkhead Box Protein P3
GPCRs: G protein-coupled receptors
GATA3: GATA Binding Protein 3
HDACs: Histone Deacetylases
HCT116: Human Colorectal Carcinoma Cell Line 116
HLA-DR: Human Leukocyte Antigen – DR isotype
anti-RG autoantibodies: IgG antibodies targeting antigens from the gut bacterium Ruminococcus gnavus (RG)
IgA: Immunoglobulin A
IgE: Immunoglobulin E
IgG1: Immunoglobulin G1
IDO: Indoleamine 2,3-dioxygenase
IFN-γ: Interferon-gamma
IRF4: Interferon Regulatory Factor 4
IL-1β: Interleukin-1 beta
IL-4: Interleukin-4
IL-10: Interleukin-10
IL-12: Interleukin-12
IL-13: Interleukin-13
IL-17: Interleukin-17
IL-23: Interleukin-23
IAP: Intestinal alkaline phosphatase
LPS: Lipopolysaccharides
LTA: Lipoteichoic acid
M1, M2: Macrophage subsets
mRNA: Messenger Ribonucleic Acid
MAMPs: Microbe-associated molecular patterns
MUC2: Mucin 2
MRL/lpr: Murphy Roths Large/Lymphoproliferative
Netosis: Neutrophil Extracellular Trap formation
NETs: Neutrophil Extracellular Traps
NLRs: NOD-like receptors
NF-κB pathway: Nuclear Factor kappa-light-chain-enhancer of activated B cells
NZBWF1 mice: New Zealand Black/White F1 hybrid mice
NZW/LacJ mice: New Zealand White/LacJ mice
PAX5: Paired Box Protein 5
PRRs: Pattern recognition receptors
PGN: Peptidoglycan
PBMCs: Peripheral blood mononuclear cells
PMA: Phorbol 12-myristate 13-acetate
PD-L1: Programmed Death-Ligand 1
ROS: Reactive Oxygen Species
RG: Ruminococcus gnavus
Tregs: Regulatory T cells
CD4+CD25+ Foxp 3+ Tregs: Regulatory T cells defined by the co-expression of: CD4, CD25, Foxp3
RORγt: Retinoic Acid Receptor-Related Orphan Receptor Gamma t
RLRs: RIG-I-like receptors
SCFAs: Short-Chain Fatty Acids
STAT3: Signal Transducer and Activator of Transcription 3
SIGNR3: Specific Intercellular adhesion molecule-3-Grabbing Non-integrin Receptor 3
SLPs: Surface proteins
SLE: Systemic Lupus Erythematosus
T-bet: T-box transcription factor TBX21
Tac: Tacrolimus
Th1, Th2, Th17: T helper cell subsets
TA: Teichoic acid
TLRs: Toll-like receptors
TGF-β: Transforming Growth Factor-beta
TNF-α: Tumor Necrosis Factor-alpha
CpG DNA: Unmethylated cytosine-phosphate-guanine DNA
VDR: Vitamin D Receptor
ZO-1: Zonula Occludens-1 (ZO-1)
A. muciniphila: Akkermansia muciniphila
B. fragilis: Bacteroides fragilis
B. bifidum: Bifidobacterium bifidum
B. longum: Bifidobacterium longum
L. acidophilus: Lactobacillus acidophilus
L. brevis: Lactobacillus brevis
L. casei: Lactobacillus casei
L. delbrueckii: Lactobacillus delbrueckii
L. fermentum: Lactobacillus fermentum
LC40: Lactobacillus fermentum CECT5716
L. plantarum: Lactobacillus plantarum
L. reuteri: Lactobacillus reuteri
L. rhamnosus: Lactobacillus rhamnosus
LGG: Lactobacillus rhamnosus GG
L. lactis: Lactococcus lactis
S. aureus: Staphylococcus aureus
S. thermophilus: Streptococcus thermophilus
